# Impact of eyesight, BMI, and the use of screen-based devices on mental well-being

**DOI:** 10.12669/pjms.40.11.10742

**Published:** 2024-12

**Authors:** Ayesha Sadiqa, Abdullah Islam, Faiza Fatima Chishti

**Affiliations:** 1 Ayesha Sadiqa, M.Phil, Ph.D Department of Physiology, CMH Lahore Medical College and Institute of Dentistry, Lahore, Pakistan National University of Medical Sciences, Rawalpindi, Pakistan; 2 Abdullah Islam, MBBS Department of Anatomy, CMH Lahore Medical College and Institute of Dentistry, Lahore, Pakistan National University of Medical Sciences, Rawalpindi, Pakistan; 3 Faiza Fatima Chishti, BDS, MSc Dental Public Health (UK) Department of Community & Preventive Dentistry, CMH Lahore Medical College and Institute of Dentistry, Lahore, Pakistan National University of Medical Sciences, Rawalpindi, Pakistan

**Keywords:** Mental health, Mental well-being, Eyesight, Hyperopia, Myopia, Smartphones

## Abstract

**Objectives:**

To observe any association between mental health and potential contributors, i.e., BMI, eyesight, and number of screen-based devices in young adults.

**Methods:**

The study proceeded after obtaining the necessary ethical approval (Ref. # 689/ERC/CMH/LMC) from the institutional ERC at CMH LMC & IOD. It was a cross-sectional study, conducted with undergraduate students aged 16-25 years from June to September 2022 at Department of Physiology at CMH Lahore Medical College and Institute of Dentistry. Those who had any history of using CNS stimulants or depressants were excluded from the study. The data was collected via a pre-designed, validated questionnaire. Sample t-test, single tail one-way ANOVA, and post-hoc Tukey’s test were used for statistical analysis, ensuring a 95% confidence interval.

**Results:**

A non-significant percentage increase in mental well-being (MWB) score of 7.09% was found in males (p=0.142). No significant difference was observed in MBW scores and BMI (p=0.129), however, underweights showed the minimum scores (38.8±7.1). In all eyesight groups, no significant difference was noticed in MBW scores (p=0.316), though those who collectively present with hyperopia and myopia expressed minimum scores (35.4±9.8). The use of different screen-based devices showed a significant association with mental health (p=0.041), while the lowest MBW scores were found in those who were using only smartphones (38.9±8.0). There was no discernible difference in MWB for daily screen time.

**Conclusion:**

Students who used only smartphones had significantly lower MWB scores than those who used smartphones and laptops. The study found low MWB scores among underweight individuals and those who were with both eye-sight issues i.e. myopia as well as hyperopia.

## INTRODUCTION

Health is not merely a state of lack of illness, rather it can be defined as a state of complete mental, physical, and social well-being.^1^ Mental well-being enables individuals to cope with stress and become more productive and responsible members of society.^2^ Numerous factors affect mental health, namely, physical, emotional, environmental, and geopolitical.^3^ Bio-researchers are studying health factors such as BMI, eyesight, and screen time. BMI is a reliable indicator of body fat and can help assess the risk of diseases related to obesity such as type 2 diabetes, hypertension, stroke, and osteoarthritis.^4^,^5^ Besides physical health implications, higher perceived weight stigma, overweight victimization,^6^,^7^ and morbidity among overweight individuals are associated with diminished mental health.^6^,^8^

There is less data available on mild visual impairment and its effect on the mental health of young people. However, some studies suggested that moderate to severe visual impairment can cause dementia in old age.^9^ In the modern era, our dependence on screen-based devices like mobiles, tablets, laptops, etc. has increased significantly. The association between time spent on screens and mental issues is also supported by literature.^10^ Too much screen time can also lead to reduced vision and a sedentary lifestyle, which can cause obesity.^4^,^11^ However, to our best knowledge, no study reflects the association of mental health with the number of devices used.

The literature revealed the discrete effect of these factors on mental health, at present our study is aimed to observe the collective effect of BMI, eyesight, and the number of screen-based devices used on mental health in young adults.

## METHODS

A cross-sectional survey-based study was carried out by the Department of Physiology at CMH Lahore Medical College and Institute of Dentistry from June to September 2022. It was a cross-sectional survey-based study involving a total of 162 first- and second-year undergraduate students in dentistry and allied health sciences.

### Ethical Approal:

The study proceeded after obtaining ethical approval (Letter Ref. # 689/ERC/CMH/LMC) from the institutional Ethical Review Committee. Consent was taken from each study participant.

Students of both genders and between 16-25 years of age were included and those who had any drug history of using CNS stimulants or depressants were excluded. To calculate Body Mass Index (BMI), the weight (kg) was divided by height (m^2^). There were three subgroups based on BMI i.e. underweight, normal, overweight/obese. Also, there were four classes were taken into account for eyesight i.e. Normal, myopic, hyperopic, and those who collectively present with both myopia as well as hyperopia. Each class of eyesight was taken in diopters. Three comparative subgroups based on BMI were studied concerning the number of screen-based devices i.e. only using smartphones, using smartphones and laptops, and those who were using tablets along with smartphones and laptops.

A pre-designed validated Questionnaire^12^ containing all close-ended questions was used as a data collection tool in the form of ‘Google Forms’. The response to each question was answered through a Likert scale from the study participants. The scores of all responses were analyzed through SPSS version-23. For descriptive analysis, averages, standard deviation, and frequency percentages were calculated. Independent Sample t-test was used to analyze the statistical difference between the averages of the two study groups, and one-way ANOVA was employed to declare the significant and non-significant difference among >2 comparative study groups. Post-hoc Tukey’s test was performed only for significant statistical values of ANOVA. P-values ≤0.05 were considered statistically significant.

## RESULTS

The male-to-female ratio was 4:23, with an insignificant 0.89% raised average BMI in males. Age-wise, there were two strata (year) i.e. 16-20 and 21-25, 47% of the participants fell in the latter category, with a 1.35% higher BMI than the rest. Sixty-five percent of participants had normal BMI scores (Table-I).

**Table-I:** Baseline statistics of the study participants.

Variables		Total n(%)	Gender			Age(years)	

			BMI* Mean±S.D	Males n(%)	Females n(%)	16-20 n(%)	21-25 n(%)
Total		162(100)	22.3±3.8	24(15)	138(85)	85(53)	77(47)
Gender	Males	24(15)	22.5±2.9	24(15)	-	11(7)	13(8)
	Females	138(85)	22.3±3.9	-	138(85)	74(45)	64(40)
Age (years)	16-20	85(53)	22.2±3.6	11(7)	74(46)	85(53)	-
	21-25	77(47)	22.5±4.0	13(8)	64(39)	-	77(47)
BMI (kg/m^2^)	Underweight	23(14)	16.7±1.4	3(2)	20(12)	15(9)	8(5)
	Normal	105(65)	21.8±1.8	17(11)	88(54)	54(33)	51(32)
	Overweight/Obese	34(21)	27.8±2.3	4(3)	30(18)	16(10)	18(11)
Eyesight	Normal	84(52)	22.1±3.9	10(6)	74(46)	44(27)	40(25)
	Myopic	40(25)	23.6±3.6	11(7)	29(18)	18(11)	22(14)
	Hyperopic	31(19)	21.0±3.3	3(2)	28(17)	21(13)	10(6)
	Both	7(4)	23.4±3.5	0	7(4)	2(1)	5(3)

* BMI = Body Mass Index.

The average BMI of underweights was 23.4% lower than that of individuals with a normal BMI. BMI scores for overweight/obese individuals were 27.5% higher than those with normal weight. Those with myopia (25%) exhibited a slight 6.7% increase in BMI compared to those with normal vision, whereas individuals with hyperopia showed a 4.9% reduction in average BMI compared to those with normal vision (Table-I).

The average MWB score of the total study population 41.4±8.6 (with S.D.) was not found significantly different from the normal distribution (p=0.115, Shapiro-Wilk), hence parametric tests were conducted. There was a significant difference (p=0.142) in the average MWB score between genders, with males showing a 7.09% higher MWB than females. Also, no statistical difference (p=0.752) was found in MWB scores between the two age groups and only a 0.96% rise was noted by the 21-25 age group compared to the 16-20 age group. In comparison to the normal BMI group, the underweight group expressed 5.8% lower, and the overweight/obese group showed a 5.58% higher MWB score, however, the difference was non-significant (p=0.129) (Table-II).

**Table-II:** Statistical comparison of the Mental Well-being score of each study variable.

Independent variables		n(%)	MWB* score Mean±S.D.*	P value
Total		162(100)	41.4±8.6
Gender	Males	24(15)	43.8±10.0	0.142
	Females	138(85)	40.9±8.4	
Age (years)	16-20	85(53)	41.2±8.4	0.752
	21-25	77(47)	41.6±8.9	
BMI (kg/m^2^)	Underweight	23(14)	38.8±7.1	0.129
	Normal	105(65)	41.2±8.7	
	Overweight/Obese	34(21)	43.5±9.1	
Eyesight (dioptres)	Normal	84(52)	41.8±8.4	0.316
	Myopic	40(25)	41.2±10.0	
	Hyperopic	31(19)	41.8±6.9	
	Both	7(4)	35.4±9.8	
Devices in use	Mobile	36(22)	38.9±8.0	0.041*
	Mobile+Laptop	102(63)	42.7±8.7	
	Mobile+Laptop+Tablet	24(15)	39.4±8.3	
Screen-time on smart-phone (hours/day)	<3	25(15)	44.3±9.0	0.133
	3–6	84(52)	40.4±8.4	
	>6	53(33)	41.5±8.6	
Screen-time on other devices (hours/day)	<3	133(82)	41.4±8.6	0.607
	3–6	22(14)	40.1±9.0	
	>6	7(4)	43.7±8.0	

* MWB = Mental Well-being, * S.D. = Standard Deviation, *p≤0.05 is statistically significant.

A non-significant difference (p=0.316) was observed among MWB scores expressed by all study groups of eyesight: normal, myopic, hyperopic, and, myopic as well as hyperopic. Compared to the normal-eyesight group, no difference was noted in MWB scores exhibited by the hyperopic group, though a reduction of 1.44% and a 15.3% was found in the MWB scores, in the myopic group and the group of individuals with myopia as well as hyperopic respectively (Table-II).

A significant difference (p=0.041) was observed in MWB scores of three comparative groups based on devices in use i.e. only mobile, mobile as well as laptop, and mobile, laptop, along with tablet. Tukey’s post-hoc test (p=0.065) revealed that this difference was seen due to “smart-phone” (average±S.D=38.9±8.0) and “smart-phone+laptop” (average±S.D.=42.7±8.7), though non-significant. The highest MWB score (42.7±8.7) was noted in the group who was using two devices (Smart-phone and laptop) and the lowest MWB score of 38.9±8.0 was found in those who were using a single device (smart-phone) (Table-II).

The comparison of MWB scores for the three groups based on daily smartphone screen time did not show a significant difference (p=0.133). The maximum MWB score was noted in those who spent <3 hours/day and a minimum was found in those who spent 3-6 hours/day on their smartphones. Here, out of a total 52% had a screen time of 3-6 hours/day and 15% had <3 hours/day on a smartphone. Again a non-significant difference (p=0.607) was noted in the MWB scores among three comparative groups concerning their screen time/day on devices other than smartphones. Here, The highest score was seen in those who spent over six hours/day on a screen other than a smartphone, while the lowest MWB score was noticed in those who spent 3-6 hours/day on a screen other than a smartphone (Table-II).

Overall, no significant difference was noted among all categories of myopia and hyperopia for MWB scores. In all the comparative eyesight groups, the highest score of 48.0±14.1 was found in students with severe myopia (>-6.00 diopters) of their left eye, and the lowest score of 39.5±9.0 was noted in those who had mild myopia (<-3.00 diopters). Only a negligible difference was present in the MWB scores of all categories of hyperopia for both the right (p=0.966) and the left eye (p=0.977) (Table-III).

**Table-III:** Statistical comparison of MWB scores among study categories of eyesight.

Eyesight categories			n (%)	MWB* Mean±S.D*	P value
Myopia (dioptres)	Eye	Total	47(100)	40.2±10.0
	Right	<-3.00	35(75)	39.8±9.4	0.637
		-3.00 to -6.00	9(19)	40.4±13.1	
		>-6.00	3(6)	43.3±9.2	
	Left	<-3.00	34(72)	39.5±9.0	0.375
		-3.00 to -6.00	11(23)	40.7±12.7	
		>-6.00	2(5)	48.0±14.1
Hyperopia (dioptres)	Total		38(100)	40.6±7.8	
	Right	<+2.25	17(45)	40.5±8.8	0.966
		+2.25 to +5.00	19(50)	41.3±7.1	
		>+5.00	2(5)	40.0±8.5	
	Left	<+2.25	22(58)	41.0±8.5	0.977
		+2.25 to +5.00	14(37)	40.7±6.8	
		>+5.00	2(5)	40.0±8.5	

* MWB = Mental Well-being, * S.D. = Standard Deviation.

## DISCUSSION

A study stated a less prevalent association between BMI and derailed mental health in their study population.^13^ Presently we also found no significant difference (p=0.129) in MWB among individuals who were underweight, normal weight, or overweight/obese. According to a study on children, BMI has little influence on the development of depression and anxiety. Rather, parental factors play an independent role in modifying these conditions. Children with symptoms of depression/anxiety tend to score lower on MWB.^14^ However, another similar study announced a causal link between MWB and BMI, according to this study, a higher BMI is associated with reduced MWB scores.^15^

Along similar lines, a study on elderly humans reported that BMI has a significant association with MWB but this relationship was non-significant in young adults.^16^ We also observed an insignificant association between age and MWB, though the age of the study participants was limited to 16-25 years. A study carried out with young adult students claimed that males had higher MWB scores than females.^17^ We also observed a non-significant 7.09% high MWB scores in male students. Another study mentioned that family-related responsibilities were the main reason for low MWB among women.^18^ Contrary to our findings another study reported that female college students scored high for psychological well-being while males scored high in terms of satisfaction and sociability.^19^

We found a non-statistical difference (p=0.316) in MWB among participants with normal eyesight, myopia, hyperopia, or myopia and hyperopia, though the lowest MWB was observed in those who had myopia as well as hyperopia. A Chinese study conducted on school students concluded that students with myopia had low MWB and tended to be depressed.^20^ However, another study carried out with college students declared a weak association between myopia and anxiety.^21^ Literature reported high mental illness scores in myopic students of Guangdong University.^22^

A study on adolescents showed that screen time had an inverse relation with mental health, those who reported excessive screen time on social media or smartphones expressed low MWB scores and had more mental health illnesses, especially in adolescent females.^23^ We noticed a significant difference (p=0.041) in MWB among those students who used one, two, or three screen-based devices, with the lowest MWB score found in those who used smartphones as the only medium of the screen. While current results did not show any noticeable difference in MWB concerning daily screen hours. Inconsistent with our findings, a study on college students concluded that high screen time is linked with reduced MBW scores.^24^

### Limitation:

A small sample size, with unequal gender proportions not only limits the generalizability of the study findings but also sways the gender-based differences in results. Moreover, factors such as sleep duration, physical activity, dietary patterns, and psychological factors may influence the MWB scores, which were not taken into account in the study.

## CONCLUSION

Students using only smartphones significantly had lower MWB scores than those using smartphones and laptops. Low MWB scores were found in underweight and in those who collectively present with myopia and hyperopia.

### Author’s Contribution:

**AS:** Conceived, designed, did statistical analysis & editing of the manuscript.

**AS** and **AI:** Did data collection, Literature search, manuscript writing.

**AS** and **FFC:** Did literature search, critical review.

All authors have approved the final manuscript and are responsible for the integrity of the research.
